# 2-(4-Chloro-3-nitro­phen­yl)-4-(4-chloro­phen­yl)-1,3-thia­zole

**DOI:** 10.1107/S1600536809039543

**Published:** 2009-10-03

**Authors:** Susanta K. Nayak, K. N. Venugopala, Deepak Chopra, Thavendran Govender, Hendrik G. Kruger, Glenn E. M. Maguire, T. N. Guru Row

**Affiliations:** a Solid State and Structural Chemistry Unit, Indian Institute of Science, Bangalore 560 012, India; b School of Chemistry, University of KwaZulu-Natal, Durban 4000, South Africa; c Department of Chemistry, Indian Institute of Science Education and Research, Bhopal 462 023, India; d School of Pharmacy and Pharmacology, University of Kwazulu-Natal, Durban 4000, South Africa

## Abstract

The title compound, C_15_H_8_Cl_2_N_2_O_2_S, crystallizes with two mol­ecules in the asymmetric unit. The dihedral angles between the 4-chloro-3-nitro­phenyl ring and the thia­zole ring are 0.5 (1) and 7.1 (1)° and those between the 4-chloro­phenyl ring and the thia­zole ring are 7.1 (1) and 7.4 (1)° in the two mol­ecules. The crystal structure is stabilized by inter­molecular C—H⋯Cl and C—H⋯O hydrogen bonds.

## Related literature

The amino­thia­zole ring system is a useful structural element in medicinal chemistry and has found broad applications in drug development, see: Fortuna *et al.* (1988 ▶); Frank *et al.* (1995 ▶); Karl *et al.* (1983 ▶); Tsuji & Ishikawa (1994 ▶).
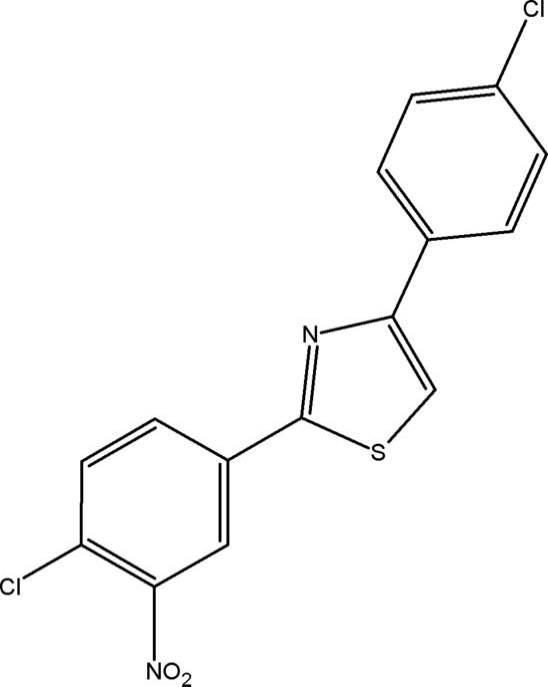


## Experimental

### 

#### Crystal data

C_15_H_8_Cl_2_N_2_O_2_S
*M* *_r_* = 351.20
Triclinic, 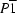

*a* = 7.4379 (19) Å
*b* = 12.305 (3) Å
*c* = 16.808 (4) Å
α = 88.596 (5)°
β = 84.131 (4)°
γ = 76.721 (5)°
*V* = 1489.3 (6) Å^3^
*Z* = 4
Mo *K*α radiation
μ = 0.58 mm^−1^
*T* = 292 K
0.28 × 0.24 × 0.15 mm

#### Data collection

Bruker SMART CCD area-detector diffractometer
Absorption correction: multi-scan (*SADABS*; Sheldrick, 1996 ▶) *T*_min_ = 0.854, *T*_max_ = 0.918
14507 measured reflections
5235 independent reflections
2855 reflections with *I* > 2σ(*I*)
*R*_int_ = 0.042

#### Refinement

*R*[*F* ^2^ > 2σ(*F* ^2^)] = 0.058
*wR*(*F* ^2^) = 0.130
*S* = 0.97
5235 reflections
397 parameters
H-atom parameters constrained
Δρ_max_ = 0.34 e Å^−3^
Δρ_min_ = −0.21 e Å^−3^

### 

Data collection: *SMART* (Bruker, 2004 ▶); cell refinement: *SAINT* (Bruker, 2004 ▶); data reduction: *SAINT*; program(s) used to solve structure: *SHELXL97* (Sheldrick, 2008 ▶); program(s) used to refine structure: *SHELXL97* (Sheldrick, 2008 ▶); molecular graphics: *ORTEP-3 for Windows* (Farrugia, 1997 ▶) and *CAMERON* (Watkin *et al.*, 1993 ▶); software used to prepare material for publication: *PLATON* (Spek, 2009 ▶).

## Supplementary Material

Crystal structure: contains datablocks global, I. DOI: 10.1107/S1600536809039543/fj2246sup1.cif

Structure factors: contains datablocks I. DOI: 10.1107/S1600536809039543/fj2246Isup2.hkl

Additional supplementary materials:  crystallographic information; 3D view; checkCIF report

## Figures and Tables

**Table 1:** Hydrogen-bond geometry (Å, °)

*D*—H⋯*A*	*D*—H	H⋯*A*	*D*⋯*A*	*D*—H⋯*A*
C11—H11⋯O1^i^	0.93	2.48	3.285 (5)	145
C15′—H15′⋯Cl2^ii^	0.93	2.73	3.610 (4)	158

Symmetry codes: (i) 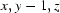; (ii) 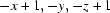.

**Fig. 1.**
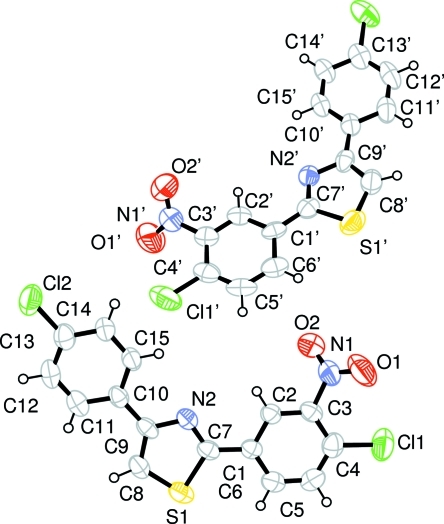
: Molecular structure shows the atom labelling Scheme with displacement ellipsoids for non-H atoms at 50% probability level, hydrogen atoms are arbitary circle.

**Fig. 2.**
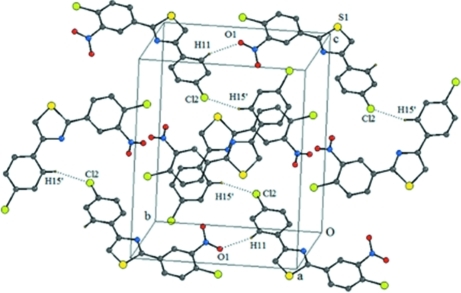
: The molecular packing depicting C—H···O and C—H···Cl intermolecular interactions in the solid state.

## supplementary crystallographic information

### Comment

The aminothiazole ring system is a useful structural element in medicinal chemistry and has found broad applications in drug development as antiallergies (Karl *et al.*, 1983), anti-inflammatory (Fortuna *et al.*, 1988), antibacterial (Tsuji *et al.*, 1994) and anti-HIV agents (Frank *et al.*, 1995]. In view of different applications of this class of compounds, we have undertaken a single-crystal structure determination of the title compound. The compound is completely planar with the nitro group not planar with the 4-chloro-4-nitrophenyl ring to avoid electrostatic repulsion with the chlorine atom in an *ortho* position, the dihedral twist being 35.4 (3)° and 48.1 (3)° respectively in the two molecules. Relevant torsion angles are given in Table 1. Figure 1 gives an *ORTEP* view depicting two molecules (A) and (B) in the asymmetric unit. The C—N bond lengths of the five-membered thiazoyl ring is different indicating that the electrnoic environment around each nitrogen atom is different. The torsion angles N2—C7—C1—C6/N2'-C7'-C1'-C6' and N2—C9—C10—C11/N2'-C9'-C10'-C11'are nearly equal to 180° indicating delocalization of the π electron density between all the three aromatic moieties, namely the thiazoyl ring and the adjacent aryl rings. The crystal structure is stabilized by C—H···O intermolecular hydrogen bonds (between molecules of the 'A' type), each of which are held by C—H···Cl intermolecular interactions (with molecules of 'B' type) between them (Figure 2).

### Experimental

A mixture of 4-chloro-3-nitrobenzonitrile, (0.1 mol), thioacetic acid (0.1 mol), boron trifluoride diethyletherate (0.1 mol) and 1,2 dichloro ethane was refluxed for 1 h at 80 C. The reaction medium was quenched with 1 N hydrochloric acid (congo red) and the obtained product i.e 4-chloro-3-nitrobenzothioamide was isolated with dichloromethane. The solvent was evaporated at reduced pressure and the crude product (Yield = 89%) left behind was recrystallized from ethyl acetate. This was taken with 2-bromo-1-(4-chlorophenyl)ethanone (0.1 mol) in absolute ethanol medium was refluxed under nitrogen atmosphere for 2 h at 80 C. Reaction medium was cooled to room temperature and poured into 50 ml of water containing sodium acetate. The precipitate obtained was filtered and recrystallized from ethanol. (Yield: 92%) and the melting point was 128–129 C.

### Refinement

All the aromatic H atoms were positioned geometrically, C—H = 0.93 Å, and refined using a riding model with *U*_iso_(H)= 1.2 *U*_eq_(C).

### Crystal data

**Table:**

C_15_H_8_Cl_2_N_2_O_2_S	*Z* = 4
*M**_r_* = 351.20	*F*(000) = 712
Triclinic, *P*1	*D*_x_ = 1.566 Mg m^−^^3^
Hall symbol: -P 1	Mo *K*α radiation, λ = 0.71073 Å
*a* = 7.4379 (19) Å	Cell parameters from 320 reflections
*b* = 12.305 (3) Å	θ = 1.0–28.0°
*c* = 16.808 (4) Å	µ = 0.58 mm^−^^1^
α = 88.596 (5)°	*T* = 292 K
β = 84.131 (4)°	Plate, colorless
γ = 76.721 (5)°	0.28 × 0.24 × 0.15 mm
*V* = 1489.3 (6) Å^3^	

### Data collection

**Table:**

Bruker SMART CCD area-detector diffractometer	5235 independent reflections
Radiation source: fine-focus sealed tube	2855 reflections with *I* > 2σ(*I*)
graphite	*R*_int_ = 0.042
φ and ω scans	θ_max_ = 25.0°, θ_min_ = 2.1°
Absorption correction: multi-scan (*SADABS*; Sheldrick, 1996)	*h* = −8→8
*T*_min_ = 0.854, *T*_max_ = 0.918	*k* = −14→14
14507 measured reflections	*l* = −19→19

### Refinement

**Table:**

Refinement on *F*^2^	Primary atom site location: structure-invariant direct methods
Least-squares matrix: full	Secondary atom site location: difference Fourier map
*R*[*F*^2^ > 2σ(*F*^2^)] = 0.058	Hydrogen site location: inferred from neighbouring sites
*wR*(*F*^2^) = 0.130	H-atom parameters constrained
*S* = 0.97	*w* = 1/[σ^2^(*F*_o_^2^) + (0.0568*P*)^2^] where *P* = (*F*_o_^2^ + 2*F*_c_^2^)/3
5235 reflections	(Δ/σ)_max_ = 0.001
397 parameters	Δρ_max_ = 0.34 e Å^−^^3^
0 restraints	Δρ_min_ = −0.20 e Å^−^^3^

### Special details

**Table:**

Geometry. All e.s.d.'s (except the e.s.d. in the dihedral angle between two l.s. planes) are estimated using the full covariance matrix. The cell e.s.d.'s are taken into account individually in the estimation of e.s.d.'s in distances, angles and torsion angles; correlations between e.s.d.'s in cell parameters are only used when they are defined by crystal symmetry. An approximate (isotropic) treatment of cell e.s.d.'s is used for estimating e.s.d.'s involving l.s. planes.
Refinement. Refinement of *F*^2^ against ALL reflections. The weighted *R*-factor *wR* and goodness of fit *S* are based on *F*^2^, conventional *R*-factors *R* are based on *F*, with *F* set to zero for negative *F*^2^. The threshold expression of *F*^2^ > σ(*F*^2^) is used only for calculating *R*-factors(gt) *etc*. and is not relevant to the choice of reflections for refinement. *R*-factors based on *F*^2^ are statistically about twice as large as those based on *F*, and *R*- factors based on ALL data will be even larger.

### Fractional atomic coordinates and isotropic or equivalent isotropic displacement parameters (Å^2^)

**Table:**

	*x*	*y*	*z*	*U*_iso_*/*U*_eq_	
S1	0.28130 (14)	−0.08178 (8)	1.08929 (6)	0.0671 (3)	
S1'	0.55762 (17)	0.57562 (10)	0.65181 (7)	0.0872 (4)	
Cl2'	1.01634 (16)	0.76240 (10)	0.20368 (8)	0.1005 (4)	
Cl2	0.02483 (18)	−0.26482 (9)	0.64160 (7)	0.0962 (4)	
Cl1'	0.55818 (19)	0.00327 (11)	0.69174 (8)	0.1110 (5)	
Cl1	0.3241 (2)	0.48445 (10)	1.11376 (8)	0.1125 (5)	
N2	0.2343 (4)	0.0036 (2)	0.95110 (18)	0.0511 (7)	
N2'	0.7301 (4)	0.4815 (2)	0.52398 (18)	0.0561 (8)	
C1	0.2750 (4)	0.1409 (3)	1.0459 (2)	0.0477 (8)	
C9	0.2261 (4)	−0.1062 (3)	0.9456 (2)	0.0501 (9)	
C10'	0.8033 (5)	0.6361 (3)	0.4402 (2)	0.0572 (10)	
C1'	0.6345 (5)	0.3473 (3)	0.6178 (2)	0.0558 (10)	
C2	0.2580 (4)	0.2243 (3)	0.9895 (2)	0.0514 (9)	
H2	0.2385	0.2088	0.9376	0.062*	
C10	0.1895 (4)	−0.1486 (3)	0.8695 (2)	0.0506 (9)	
C7	0.2621 (4)	0.0289 (3)	1.0228 (2)	0.0497 (9)	
C9'	0.7236 (5)	0.5945 (3)	0.5149 (2)	0.0582 (10)	
C3	0.2695 (5)	0.3300 (3)	1.0087 (2)	0.0551 (9)	
N1	0.2537 (5)	0.4131 (3)	0.9443 (3)	0.0807 (11)	
C3'	0.6725 (5)	0.1531 (4)	0.5877 (2)	0.0627 (10)	
C2'	0.6890 (5)	0.2590 (3)	0.5648 (2)	0.0575 (10)	
H2'	0.7373	0.2712	0.5129	0.069*	
C4	0.3003 (5)	0.3551 (3)	1.0859 (3)	0.0650 (10)	
C12	0.1416 (5)	−0.2967 (3)	0.7884 (3)	0.0694 (11)	
H12	0.1406	−0.3715	0.7822	0.083*	
C15'	0.8695 (5)	0.5650 (3)	0.3768 (2)	0.0627 (10)	
H15'	0.8679	0.4899	0.3830	0.075*	
C13	0.0966 (5)	−0.2213 (3)	0.7275 (2)	0.0657 (11)	
C6'	0.5613 (5)	0.3250 (4)	0.6947 (2)	0.0728 (12)	
H6'	0.5253	0.3824	0.7320	0.087*	
C5'	0.5417 (5)	0.2198 (4)	0.7162 (2)	0.0770 (13)	
H5'	0.4892	0.2076	0.7673	0.092*	
C15	0.1492 (5)	−0.0769 (3)	0.8060 (2)	0.0617 (10)	
H15	0.1539	−0.0025	0.8110	0.074*	
C14	0.1024 (5)	−0.1121 (3)	0.7358 (2)	0.0692 (11)	
H14	0.0748	−0.0619	0.6940	0.083*	
C11	0.1880 (5)	−0.2598 (3)	0.8587 (3)	0.0663 (11)	
H11	0.2191	−0.3106	0.8998	0.080*	
C4'	0.5983 (5)	0.1320 (4)	0.6634 (3)	0.0704 (11)	
C6	0.3070 (5)	0.1658 (3)	1.1221 (2)	0.0633 (10)	
H6	0.3202	0.1102	1.1610	0.076*	
C7'	0.6514 (5)	0.4589 (3)	0.5922 (2)	0.0577 (10)	
C14'	0.9381 (5)	0.6015 (3)	0.3042 (3)	0.0681 (11)	
H14'	0.9827	0.5518	0.2621	0.082*	
N1'	0.7310 (6)	0.0637 (3)	0.5286 (3)	0.0868 (11)	
C13'	0.9395 (5)	0.7127 (4)	0.2952 (3)	0.0708 (11)	
C5	0.3194 (5)	0.2702 (4)	1.1411 (2)	0.0697 (11)	
H5	0.3415	0.2846	1.1929	0.084*	
C8	0.2493 (5)	−0.1640 (3)	1.0153 (2)	0.0597 (10)	
H8	0.2477	−0.2391	1.0214	0.072*	
C12'	0.8791 (6)	0.7851 (3)	0.3577 (3)	0.0791 (13)	
H12'	0.8836	0.8598	0.3512	0.095*	
O2'	0.6821 (5)	0.0812 (3)	0.4624 (2)	0.1199 (13)	
C11'	0.8114 (5)	0.7484 (3)	0.4306 (3)	0.0734 (12)	
H11'	0.7713	0.7980	0.4731	0.088*	
O1'	0.8319 (6)	−0.0236 (3)	0.5481 (2)	0.1366 (15)	
O2	0.3185 (6)	0.3817 (3)	0.8786 (2)	0.1309 (15)	
C8'	0.6340 (6)	0.6561 (3)	0.5786 (3)	0.0785 (12)	
H8'	0.6164	0.7332	0.5819	0.094*	
O1	0.1819 (6)	0.5084 (3)	0.9591 (2)	0.1363 (15)	

### Atomic displacement parameters (Å^2^)

**Table:**

	*U*^11^	*U*^22^	*U*^33^	*U*^12^	*U*^13^	*U*^23^
S1	0.0729 (7)	0.0615 (7)	0.0633 (7)	−0.0124 (5)	−0.0007 (5)	0.0185 (5)
S1'	0.0982 (9)	0.0881 (9)	0.0658 (7)	−0.0012 (7)	−0.0043 (6)	−0.0207 (6)
Cl2'	0.0947 (9)	0.0892 (8)	0.1212 (10)	−0.0332 (7)	−0.0096 (7)	0.0432 (8)
Cl2	0.1247 (10)	0.0670 (7)	0.0990 (9)	−0.0185 (7)	−0.0239 (8)	−0.0217 (6)
Cl1'	0.1280 (11)	0.1157 (10)	0.1013 (10)	−0.0510 (9)	−0.0264 (8)	0.0499 (8)
Cl1	0.1490 (12)	0.0793 (8)	0.1167 (11)	−0.0433 (8)	−0.0024 (9)	−0.0282 (7)
N2	0.0489 (18)	0.0452 (18)	0.057 (2)	−0.0088 (14)	−0.0001 (15)	0.0068 (15)
N2'	0.0536 (19)	0.056 (2)	0.058 (2)	−0.0087 (15)	−0.0144 (16)	−0.0017 (16)
C1	0.041 (2)	0.052 (2)	0.048 (2)	−0.0085 (17)	0.0027 (16)	0.0034 (18)
C9	0.038 (2)	0.045 (2)	0.063 (3)	−0.0075 (17)	0.0035 (17)	0.0139 (19)
C10'	0.050 (2)	0.050 (2)	0.073 (3)	−0.0085 (18)	−0.022 (2)	0.001 (2)
C1'	0.046 (2)	0.082 (3)	0.038 (2)	−0.008 (2)	−0.0140 (17)	0.002 (2)
C2	0.054 (2)	0.053 (2)	0.049 (2)	−0.0172 (18)	−0.0032 (17)	0.0029 (19)
C10	0.047 (2)	0.043 (2)	0.060 (2)	−0.0117 (17)	0.0071 (18)	0.0029 (19)
C7	0.041 (2)	0.054 (2)	0.051 (2)	−0.0083 (17)	0.0048 (17)	0.0083 (18)
C9'	0.054 (2)	0.053 (3)	0.067 (3)	−0.0032 (19)	−0.021 (2)	−0.013 (2)
C3	0.051 (2)	0.055 (2)	0.057 (3)	−0.0135 (18)	0.0016 (18)	0.011 (2)
N1	0.105 (3)	0.057 (3)	0.082 (3)	−0.028 (2)	0.002 (2)	0.004 (2)
C3'	0.060 (3)	0.074 (3)	0.055 (3)	−0.015 (2)	−0.010 (2)	0.009 (2)
C2'	0.050 (2)	0.073 (3)	0.047 (2)	−0.010 (2)	−0.0025 (18)	0.009 (2)
C4	0.058 (2)	0.063 (3)	0.072 (3)	−0.014 (2)	0.008 (2)	−0.012 (2)
C12	0.068 (3)	0.044 (2)	0.097 (3)	−0.016 (2)	0.000 (2)	−0.008 (2)
C15'	0.062 (3)	0.044 (2)	0.083 (3)	−0.0150 (19)	−0.011 (2)	0.005 (2)
C13	0.063 (3)	0.052 (3)	0.079 (3)	−0.010 (2)	0.002 (2)	−0.007 (2)
C6'	0.067 (3)	0.095 (3)	0.054 (3)	−0.011 (2)	−0.014 (2)	0.004 (2)
C5'	0.070 (3)	0.116 (4)	0.046 (3)	−0.022 (3)	−0.009 (2)	0.023 (3)
C15	0.078 (3)	0.044 (2)	0.064 (3)	−0.021 (2)	0.005 (2)	0.000 (2)
C14	0.098 (3)	0.046 (2)	0.065 (3)	−0.021 (2)	−0.001 (2)	−0.003 (2)
C11	0.062 (3)	0.048 (2)	0.087 (3)	−0.0114 (19)	−0.004 (2)	0.008 (2)
C4'	0.064 (3)	0.086 (3)	0.066 (3)	−0.022 (2)	−0.021 (2)	0.025 (3)
C6	0.068 (3)	0.065 (3)	0.056 (3)	−0.016 (2)	−0.003 (2)	0.010 (2)
C7'	0.049 (2)	0.066 (3)	0.056 (3)	−0.0033 (19)	−0.0160 (19)	−0.003 (2)
C14'	0.065 (3)	0.054 (3)	0.085 (3)	−0.018 (2)	−0.004 (2)	0.010 (2)
N1'	0.108 (3)	0.070 (3)	0.082 (3)	−0.021 (2)	−0.009 (3)	0.006 (3)
C13'	0.053 (3)	0.062 (3)	0.099 (3)	−0.013 (2)	−0.015 (2)	0.007 (3)
C5	0.074 (3)	0.082 (3)	0.053 (3)	−0.019 (2)	−0.003 (2)	−0.008 (2)
C8	0.060 (2)	0.045 (2)	0.072 (3)	−0.0136 (18)	0.004 (2)	0.007 (2)
C12'	0.069 (3)	0.049 (3)	0.127 (4)	−0.022 (2)	−0.030 (3)	0.020 (3)
O2'	0.169 (4)	0.100 (3)	0.089 (3)	−0.018 (2)	−0.032 (2)	−0.013 (2)
C11'	0.074 (3)	0.051 (3)	0.099 (4)	−0.012 (2)	−0.029 (3)	−0.008 (2)
O1'	0.181 (4)	0.076 (2)	0.131 (3)	0.013 (2)	−0.015 (3)	0.011 (2)
O2	0.237 (5)	0.094 (3)	0.074 (2)	−0.070 (3)	−0.002 (3)	0.011 (2)
C8'	0.093 (3)	0.061 (3)	0.082 (3)	−0.012 (2)	−0.023 (3)	−0.007 (2)
O1	0.185 (4)	0.066 (2)	0.143 (3)	−0.014 (2)	0.011 (3)	0.028 (2)

### Geometric parameters (Å, °)

**Table:**

S1—C8	1.692 (4)	C3'—C4'	1.377 (5)
S1—C7	1.730 (3)	C3'—C2'	1.378 (5)
S1'—C8'	1.695 (4)	C3'—N1'	1.459 (5)
S1'—C7'	1.737 (4)	C2'—H2'	0.9300
Cl2'—C13'	1.733 (4)	C4—C5	1.373 (5)
Cl2—C13	1.727 (4)	C12—C13	1.379 (5)
Cl1'—C4'	1.726 (4)	C12—C11	1.380 (5)
Cl1—C4	1.723 (4)	C12—H12	0.9300
N2—C7	1.300 (4)	C15'—C14'	1.380 (5)
N2—C9	1.372 (4)	C15'—H15'	0.9300
N2'—C7'	1.288 (4)	C13—C14	1.366 (5)
N2'—C9'	1.385 (4)	C6'—C5'	1.370 (5)
C1—C2	1.372 (4)	C6'—H6'	0.9300
C1—C6	1.380 (5)	C5'—C4'	1.376 (5)
C1—C7	1.468 (5)	C5'—H5'	0.9300
C9—C8	1.361 (5)	C15—C14	1.371 (5)
C9—C10	1.467 (5)	C15—H15	0.9300
C10'—C15'	1.372 (5)	C14—H14	0.9300
C10'—C11'	1.402 (5)	C11—H11	0.9300
C10'—C9'	1.465 (5)	C6—C5	1.357 (5)
C1'—C2'	1.383 (5)	C6—H6	0.9300
C1'—C6'	1.396 (5)	C14'—C13'	1.374 (5)
C1'—C7'	1.458 (5)	C14'—H14'	0.9300
C2—C3	1.372 (4)	N1'—O2'	1.205 (4)
C2—H2	0.9300	N1'—O1'	1.219 (4)
C10—C15	1.380 (5)	C13'—C12'	1.366 (5)
C10—C11	1.388 (5)	C5—H5	0.9300
C9'—C8'	1.352 (5)	C8—H8	0.9300
C3—C4	1.395 (5)	C12'—C11'	1.382 (5)
C3—N1	1.464 (5)	C12'—H12'	0.9300
N1—O1	1.193 (4)	C11'—H11'	0.9300
N1—O2	1.195 (4)	C8'—H8'	0.9300
			
C8—S1—C7	89.27 (18)	C14—C13—Cl2	119.9 (3)
C8'—S1'—C7'	89.0 (2)	C12—C13—Cl2	119.5 (3)
C7—N2—C9	111.8 (3)	C5'—C6'—C1'	121.2 (4)
C7'—N2'—C9'	112.2 (3)	C5'—C6'—H6'	119.4
C2—C1—C6	118.2 (3)	C1'—C6'—H6'	119.4
C2—C1—C7	119.1 (3)	C6'—C5'—C4'	121.1 (4)
C6—C1—C7	122.7 (3)	C6'—C5'—H5'	119.4
C8—C9—N2	113.8 (3)	C4'—C5'—H5'	119.4
C8—C9—C10	126.9 (3)	C14—C15—C10	122.0 (4)
N2—C9—C10	119.3 (3)	C14—C15—H15	119.0
C15'—C10'—C11'	118.2 (4)	C10—C15—H15	119.0
C15'—C10'—C9'	120.1 (3)	C13—C14—C15	119.5 (4)
C11'—C10'—C9'	121.7 (4)	C13—C14—H14	120.2
C2'—C1'—C6'	117.4 (4)	C15—C14—H14	120.2
C2'—C1'—C7'	120.0 (3)	C12—C11—C10	121.5 (4)
C6'—C1'—C7'	122.5 (4)	C12—C11—H11	119.3
C3—C2—C1	120.9 (3)	C10—C11—H11	119.3
C3—C2—H2	119.5	C5'—C4'—C3'	118.1 (4)
C1—C2—H2	119.5	C5'—C4'—Cl1'	119.0 (4)
C15—C10—C11	117.3 (4)	C3'—C4'—Cl1'	122.7 (4)
C15—C10—C9	120.3 (3)	C5—C6—C1	121.0 (4)
C11—C10—C9	122.3 (3)	C5—C6—H6	119.5
N2—C7—C1	123.5 (3)	C1—C6—H6	119.5
N2—C7—S1	113.8 (3)	N2'—C7'—C1'	124.4 (3)
C1—C7—S1	122.7 (3)	N2'—C7'—S1'	113.7 (3)
C8'—C9'—N2'	113.3 (4)	C1'—C7'—S1'	121.9 (3)
C8'—C9'—C10'	126.8 (4)	C13'—C14'—C15'	118.8 (4)
N2'—C9'—C10'	119.9 (3)	C13'—C14'—H14'	120.6
C2—C3—C4	120.6 (3)	C15'—C14'—H14'	120.6
C2—C3—N1	117.2 (3)	O2'—N1'—O1'	123.4 (4)
C4—C3—N1	122.2 (4)	O2'—N1'—C3'	118.3 (4)
O1—N1—O2	122.6 (4)	O1'—N1'—C3'	118.3 (4)
O1—N1—C3	119.8 (4)	C12'—C13'—C14'	120.7 (4)
O2—N1—C3	117.6 (4)	C12'—C13'—Cl2'	119.3 (3)
C4'—C3'—C2'	121.3 (4)	C14'—C13'—Cl2'	120.0 (4)
C4'—C3'—N1'	120.5 (4)	C6—C5—C4	121.7 (4)
C2'—C3'—N1'	118.2 (4)	C6—C5—H5	119.2
C3'—C2'—C1'	120.9 (4)	C4—C5—H5	119.2
C3'—C2'—H2'	119.6	C9—C8—S1	111.3 (3)
C1'—C2'—H2'	119.6	C9—C8—H8	124.4
C5—C4—C3	117.5 (4)	S1—C8—H8	124.4
C5—C4—Cl1	119.0 (3)	C13'—C12'—C11'	120.5 (4)
C3—C4—Cl1	123.4 (3)	C13'—C12'—H12'	119.8
C13—C12—C11	119.1 (4)	C11'—C12'—H12'	119.8
C13—C12—H12	120.4	C12'—C11'—C10'	119.7 (4)
C11—C12—H12	120.4	C12'—C11'—H11'	120.1
C10'—C15'—C14'	122.1 (4)	C10'—C11'—H11'	120.1
C10'—C15'—H15'	119.0	C9'—C8'—S1'	111.7 (3)
C14'—C15'—H15'	119.0	C9'—C8'—H8'	124.1
C14—C13—C12	120.5 (4)	S1'—C8'—H8'	124.1
			
C7—N2—C9—C8	−0.2 (4)	C9—C10—C15—C14	−175.7 (3)
C7—N2—C9—C10	178.1 (3)	C12—C13—C14—C15	−1.5 (6)
C6—C1—C2—C3	−1.1 (5)	Cl2—C13—C14—C15	176.2 (3)
C7—C1—C2—C3	−179.9 (3)	C10—C15—C14—C13	−0.5 (6)
C8—C9—C10—C15	173.0 (3)	C13—C12—C11—C10	0.3 (6)
N2—C9—C10—C15	−5.1 (5)	C15—C10—C11—C12	−2.3 (5)
C8—C9—C10—C11	−5.0 (5)	C9—C10—C11—C12	175.8 (3)
N2—C9—C10—C11	176.9 (3)	C6'—C5'—C4'—C3'	−1.1 (6)
C9—N2—C7—C1	−179.4 (3)	C6'—C5'—C4'—Cl1'	−176.4 (3)
C9—N2—C7—S1	0.2 (4)	C2'—C3'—C4'—C5'	−0.4 (5)
C2—C1—C7—N2	−0.3 (5)	N1'—C3'—C4'—C5'	−178.6 (4)
C6—C1—C7—N2	−179.2 (3)	C2'—C3'—C4'—Cl1'	174.7 (3)
C2—C1—C7—S1	−179.9 (2)	N1'—C3'—C4'—Cl1'	−3.6 (5)
C6—C1—C7—S1	1.3 (4)	C2—C1—C6—C5	0.7 (5)
C8—S1—C7—N2	−0.1 (3)	C7—C1—C6—C5	179.5 (3)
C8—S1—C7—C1	179.5 (3)	C9'—N2'—C7'—C1'	178.9 (3)
C7'—N2'—C9'—C8'	−1.1 (4)	C9'—N2'—C7'—S1'	1.0 (4)
C7'—N2'—C9'—C10'	−178.9 (3)	C2'—C1'—C7'—N2'	−6.4 (5)
C15'—C10'—C9'—C8'	−171.7 (4)	C6'—C1'—C7'—N2'	174.8 (3)
C11'—C10'—C9'—C8'	7.1 (6)	C2'—C1'—C7'—S1'	171.4 (3)
C15'—C10'—C9'—N2'	5.8 (5)	C6'—C1'—C7'—S1'	−7.5 (5)
C11'—C10'—C9'—N2'	−175.5 (3)	C8'—S1'—C7'—N2'	−0.6 (3)
C1—C2—C3—C4	0.6 (5)	C8'—S1'—C7'—C1'	−178.5 (3)
C1—C2—C3—N1	178.8 (3)	C10'—C15'—C14'—C13'	−0.2 (5)
C2—C3—N1—O1	146.7 (4)	C4'—C3'—N1'—O2'	132.4 (4)
C4—C3—N1—O1	−35.2 (6)	C2'—C3'—N1'—O2'	−45.9 (6)
C2—C3—N1—O2	−35.0 (5)	C4'—C3'—N1'—O1'	−50.1 (6)
C4—C3—N1—O2	143.1 (4)	C2'—C3'—N1'—O1'	131.7 (4)
C4'—C3'—C2'—C1'	1.2 (5)	C15'—C14'—C13'—C12'	1.9 (6)
N1'—C3'—C2'—C1'	179.5 (3)	C15'—C14'—C13'—Cl2'	−177.5 (3)
C6'—C1'—C2'—C3'	−0.5 (5)	C1—C6—C5—C4	0.2 (6)
C7'—C1'—C2'—C3'	−179.4 (3)	C3—C4—C5—C6	−0.7 (6)
C2—C3—C4—C5	0.3 (5)	Cl1—C4—C5—C6	−178.3 (3)
N1—C3—C4—C5	−177.8 (3)	N2—C9—C8—S1	0.2 (4)
C2—C3—C4—Cl1	177.7 (3)	C10—C9—C8—S1	−178.0 (3)
N1—C3—C4—Cl1	−0.3 (5)	C7—S1—C8—C9	0.0 (3)
C11'—C10'—C15'—C14'	−1.7 (5)	C14'—C13'—C12'—C11'	−1.6 (6)
C9'—C10'—C15'—C14'	177.1 (3)	Cl2'—C13'—C12'—C11'	177.8 (3)
C11—C12—C13—C14	1.6 (6)	C13'—C12'—C11'—C10'	−0.5 (6)
C11—C12—C13—Cl2	−176.1 (3)	C15'—C10'—C11'—C12'	2.1 (5)
C2'—C1'—C6'—C5'	−1.0 (5)	C9'—C10'—C11'—C12'	−176.7 (3)
C7'—C1'—C6'—C5'	177.9 (3)	N2'—C9'—C8'—S1'	0.7 (4)
C1'—C6'—C5'—C4'	1.9 (6)	C10'—C9'—C8'—S1'	178.2 (3)
C11—C10—C15—C14	2.4 (5)	C7'—S1'—C8'—C9'	−0.1 (3)

### Hydrogen-bond geometry (Å, °)

**Table:**

*D*—H···*A*	*D*—H	H···*A*	*D*···*A*	*D*—H···*A*
C11—H11···O1^i^	0.93	2.48	3.285 (5)	145
C15'—H15'···Cl2^ii^	0.93	2.73	3.610 (4)	158

Symmetry codes: (i) *x*, *y*−1, *z*; (ii) −*x*+1, −*y*, −*z*+1.
